# The impact of viewing thinness and fitness transformation images on women’s body dissatisfaction, weight management intentions, and emotions - an ecological momentary assessment study

**DOI:** 10.1192/j.eurpsy.2025.496

**Published:** 2025-08-26

**Authors:** S. Pan, X. Zeng, Y. Huang, Q. Sun, J. He

**Affiliations:** 1 Department of Psychiatry, The Chinese University of Hong Kong, Hong Kong, Hong Kong; 2 Department of Psychology, Central South University, Changsha, China; 3 Department of Psychology, The Chinese University of Hong Kong, Hong Kong, Hong Kong; 4 Department of Humanities and Social Science , The Chinese University of Hong Kong, Shenzhen, Shenzhen, China

## Abstract

**Introduction:**

Transformation images, widely circulated on social media, depict a contrast between an ‘unideal’ body state and an ‘ideal’ one, often categorized under ‘thinspiration’ or ‘fitspiration’. Unlike ‘ideal’ images shown individually, transformation images not only showcase an ‘ideal’ physique but also suggest its attainability. Although research has found that exposure to ‘ideal’ images is linked to risk factors for eating disorders, scant research has focused on the effects of transformation images.

**Objectives:**

This study aims to investigate the impact of viewing transformation images on body dissatisfaction, weight management intentions, and emotions in adult young women.

**Methods:**

137 Chinese adult women (mean age 21.7±3.0 years) were randomly assigned to one of four image conditions: side-by-side thinness transformation images, side-by-side fitness transformation images, thinness images shown individually, and fitness images shown individually. After baseline assessment of Body Dissatisfaction, Body Mass Index, Internalization of Thinness/Muscularity, participants underwent a 7-day Ecological Momentary Assessment. Prompts were delivered four times per day at 3 to 4-hour intervals. Each prompt included the display of an image for 5 seconds, followed by a survey assessing body dissatisfaction, weight management intentions (intentions to diet, binge eating, and work out), and emotions (positive and negative emotions). Linear mixed models were used to analyze the data, controlling for baseline assessments, autocorrelation, and within-person variability.

**Results:**

Fitness transformation images significantly increased intentions to diet (*p* < 0.05), binge eating (*p* < 0.01), and work out (*p* < 0.05) compared to fitness images shown individually (Table 1). However, this impact was not observed with thinness transformation images (Table 2). Neither thinness nor fitness transformation images had a significant impact on body dissatisfaction or emotions.

**Image 1:**

**Figure:**
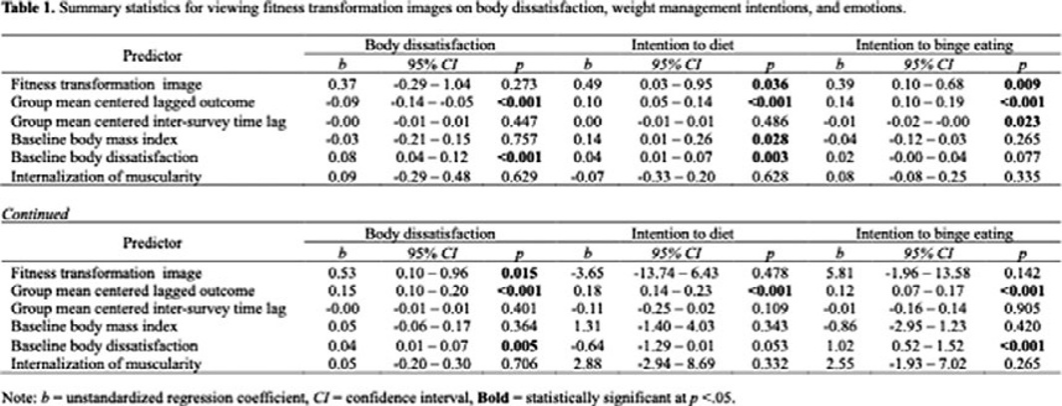

**Image 2:**

**Figure:**
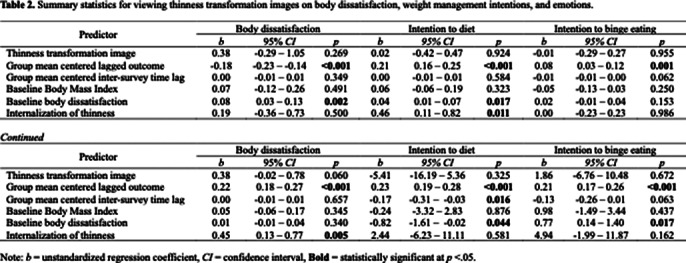

**Conclusions:**

Our study demonstrates that fitness transformation images have a stronger impact on weight management intentions than fitness images presented individually. This finding aligns with the cultural shift in the ideal of physical attractiveness, which now emphasizes ‘fitness’ and a ‘healthy lifestyle’ over ‘thinness’ in women. It is essential to focus on the exposure fitness transformation images on social media and to develop strategies for mitigating potential negative effects in the future.

**Disclosure of Interest:**

S. Pan: None Declared, X. Zeng: None Declared, Y. Huang: None Declared, Q. Sun: None Declared, J. He Grant / Research support from: This research was partially supported by the Presidential Fund of the Chinese University of Hong Kong, Shenzhen, to Jinbo He (Grant Number: PF.01.001428)

